# Membrane Bioincompatibility and Ultrafiltration Effects on Pulse Wave Analysis during Haemodialysis

**DOI:** 10.5402/2013/892315

**Published:** 2012-12-17

**Authors:** Maria-Pau Valenzuela, Jaume Almirall, María-José Amengual

**Affiliations:** ^1^Nephrology Service, UDIAT, Corporació Parc Taulí and Departament de Medicina, Institut Universitari Parc Taulí (UAB), Parc Taulí S/N, 08208 Sabadell, Spain; ^2^Laboratory Department, UDIAT, Corporació Parc Taulí and Departament de Medicina, Institut Universitari Parc Taulí (UAB), Parc Taulí S/N, 08208 Sabadell, Spain

## Abstract

Membrane bioincompatibility was demonstrated by successive white blood cell counts and C3a generation. Pulse wave analysis was obtained by applanation tonometry (SphygmoCor) in a sequential way: basal, after 30 minutes with nul ultrafiltration, and after a complete dialysis with ultrafiltration. At 15 minutes of haemodialysis, significant decrease in leukocyte count occurred: 6801 ± 1186 versus 4412 ± 1333 (*P* < 0.001), while C3a levels sharply increased from 427 ± 269 to 3501 ± 1638 ng/mL (*P* < 0.000). No changes were observed in augmentation index without ultrafiltration: 26.1 ± 11.1 versus 26.6 ± 12.4. Only aortic systolic blood pressure was lower at 15 minutes: 120.1 ± 17.7 versus 110.4 ± 25.8 mmHg (*P* = 0.009), in agreement with a reduction in brachial systolic blood pressure: 135.1 ± 18.1 versus 122.7 ± 27.4 mmHg (*P* = 0.01), without changes in aortic or brachial diastolic blood pressure. Important changes in pulse wave analysis were observed after a complete haemodialysis session: augmentation index 29.9 ± 10.1 versus 18.6 ± 15.0, aortic systolic blood pressure 139.8 ± 25.5 versus 119.4 ± 28.5 mmHg (*P* < 0.00), without changes in aortic diastolic blood pressure. In summary, haemodialysis with cellulose diacetate acutely induced a transient state of immunoactivation due to bioincompatibility, this phenomenon was nondetectable by pulse wave analysis. Complete haemodialysis session led to important changes in pulse wave analysis.

## 1. Introduction

Cardiovascular (CV) disease is the main cause of morbidity and mortality in patients with end-stage renal disease (ESRD). The increased risk is partly due to a higher prevalence of traditional CV risk factors. Nevertheless, these patients also present other nontraditional CV risk factors related with the setting of uremic background [1] that result in functional and structural alterations of the arterial wall, leading to an increase in arterial stiffness. As in the general population, arterial stiffening has been described as an independent predictor of both CV and overall mortality in haemodialysis (HD) patients [2–8].

Another component that has been proposed to play a role in CV risk is the HD session per se. The dialysis procedure may induce acute functional alterations of the arterial wall through several mechanisms, the most remarkable being the intermittent immunoactivation state induced by dialysis [9–11], and the acute intravascular volume drop produced during the HD session [12–17]. It has been proposed that these acute functional alterations could be detected by noninvasive pulse wave analysis (PWA) [12].

As PWA measurements are being increasingly introduced in the clinical setting, the main objective of our study was to separately analyze the acute effects on PWA of bioincompatibility and ultrafiltration (UF) during the dialysis session.

## 2. Subjects and Methods

### 2.1. Study Population

We separately analyzed the acute effect on PWA of membrane bioincompatibility (Study I, *n* = 11) and ultrafiltration (Study II, *n* = 19) during the HD session. Patients were eligible for entry into the study when (1) they had been on HD for at least 3 months, (2) they were in a stable clinical situation, and (3) they did not suffer cardiovascular instability on dialysis. All selected patients agreed to participate in the study, which was approved by the Hospital Ethics Committee. 

### 2.2. Pulse Wave Analysis (PWA)

Radial arterial waveform in the nonfistula arm was measured by applanation tonometry with the SphygmoCor device, using the PWA software package. Studied variables included augmentation index corrected by heart rate (AI@75), subendocardial viability ratio (SEVR), ejection duration (ED), and aortic systolic and diastolic blood pressure. Measurements were taken by a single observer in triplicate and averaged.

### 2.3. Study I: Membrane Bioincompatibility Effects

As a biological demonstration of membrane bioincompatibility, successive white blood cell counts in the ADVIA autoanalyzer and C3a generation (enzyme immunoassay Quidel) were obtained. The analysis was performed by leukocyte count and C3a levels monitored before, at 15, 30, 60 minutes, and at the end of the HD session. PWA was performed by SphygmoCor system before, at 15 and 30 minutes of starting HD session. All patients were dialyzed with the same cellulose diacetate membrane (Baxter) with surfaces of 170 or 210 m^2^ and dialysate calcium concentrations of 1.5 mM. In order to avoid the effects of intravascular volume changes on PWA, fluid removal was completely avoided (ultrafiltration = 0) during the 30 minutes of PWA monitoring.

### 2.4. Study II: UF Effects

PWA was performed before and after a conventional HD session with the same cellulose diacetate membrane (Baxter) and dialysate calcium concentrations than in Study I. The procedure was carried out in normal conditions, with programmed UF as clinically needed based on their dry weight. A total of 19 patients were studied, and the mean ultrafiltration was 2.43 ± 1.12 Kg.

### 2.5. Statistical Analyses

Statistical analysis was performed using the computer software SPSS 17 for Windows. Data are expressed as mean ± SD. The effect of HD session on the measured variables was tested by means of the paired *t*-test and repeated measures ANOVA. Univariate correlations between variables were assessed using the Pearson's coefficient of correlation test. *P* < 0.05 was considered statistically significant.

## 3. Results

### 3.1. Study I: Membrane Bioincompatibility Effects

Eleven patients were included in Study I, with a mean age of 76.9 ± 10.5 years, 54.6% men, with a mean time on dialysis of 33.9 ± 37.9 months. Mean predialysis peripheral blood pressure measured in triplicate and averaged was 135.1 ± 18.1/61.7 ± 7.5 mmHg.

Membrane bioincompatibility results related with leukocyte count, C3a, peripheral and central blood pressure and stiffness parameters are displayed in Table 1. A significant decrease in leukocyte count occurred at the beginning of dialysis: 6801 ± 1186 versus 4412 ± 1333 at 15 minutes (*P* < 0.001), while C3a levels sharply increased from 427 ± 269 to 3501 ± 1638 ng/mL at 15 minutes (*P* < 0.000), both parameters being inversely correlated (*r* ^2^ = 0.74, *P* = 0.01). No changes were demonstrated in any of the variables analysed in PWA: AI@75 (26.1 ± 11.1 versus 26.6 ± 12.4), ED (37.1 ± 5.9 versus 35.8 ± 5.1), and SEVR (129.1 ± 30.3 versus 141.1 ± 29.5). Only aortic systolic BP was lower at 15 minutes: 120.1 ± 17.7 versus 110.4 ± 25.8  mmHg (*P* = 0.009), in correlation with a reduction of brachial systolic BP: 135.1 ± 18.1 versus 122.7 ± 27.4 mmHg (*P* = 0.01), without changes in aortic or brachial diastolic BP.

### 3.2. Study II: UF Effects

Nineteen patients were included in Study II, with a mean age of 71.4 ± 12.2 years, 63.2% were men, and the mean time on dialysis was 36.2 ± 47.4 months. Mean predialysis brachial BP was 153.7 ± 26.7/74.7 ± 13.3 mmHg. Mean UF was 2.43 ± 1.12 Kg.

**Figure 1:**
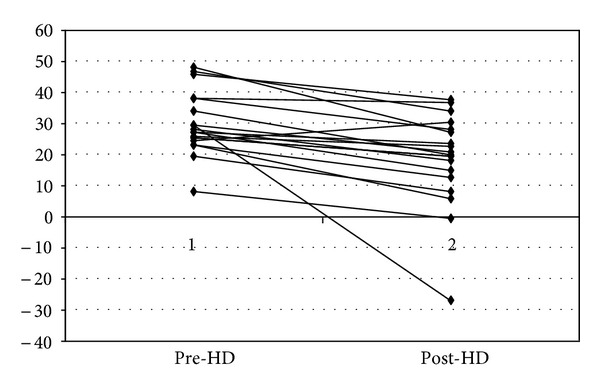
Dialysis effect on augmentation index.

UF effects results are displayed in Table 2. Important changes in all the PWA parameters evaluated were observed at the end of the haemodialysis session: AI 29.9 ± 10.1 versus 18.6 ± 15.0 (Figure 1), ED 37.6 ± 3.6 versus 32.8 ± 4.6, SEVR 124.6 ± 19.9 versus 171.7 ± 37.1, central aortic systolic BP 139.8 ± 25.5 versus 119.4 ± 28.5 mmHg (all *P* < 0.00), without changes in aortic diastolic BP 75.8 ± 13.9 versus 75.1 ± 17.9 mmHg (p : ns). However, there was no good correlation between the amount ultrafiltered and the changes in PWA parameters (*r* = 0.65), suggesting that arterial stiffness improvement was not caused by fluid removal alone.

## 4. Discussion

Arterial stiffness measurement in ESRD patients has gained importance in the last years, especially for its relationship with increased left ventricular hypertrophy and decreased coronary perfusion during diastole [12]. Nevertheless, the acute effects of the HD session on arterial stiffness have been sparsely studied. The results of previous publications are controversial, and it is not clear whether HD per se can induce acute functional changes in the arterial wall [18, 19]. This study was designed to separately evaluate the two seemingly and opposite main determinants of arterial stiffness changes during the HD session. In Study I, we demonstrate the existence of immunoactivation assessed by considerable changes in leukocyte count and C3 levels. However, such bioincompatibility was not detectable by PWA, since we did not observe any changes in any arterial stiffness parameters. Previous studies have shown changes in vascular function using other functional studies as pulse wave velocity (PWV), flow-mediated dilation, or endothelium-independent vasodilation [9, 10]. To our knowledge, no previous studies have analyzed the effect of the membrane bioincompatibility on AI@75.

When the dialysis procedure was performed with the normal pattern of UF, and PWA was measured at the end of the dialysis session (Study II), a significant effect on arterial stiffness parameters was shown. We observed a decrease in both aortic systolic BP and AI@75 at the end of the HD session. Contrary to expected, we found no correlation between changes in arterial stiffness parameters and the magnitude of fluid removal. Previous publications have analysed the effect of the HD session on arterial stiffness yielding different results. Some of them defend a lack of improvement of arterial stiffness arguing that vascular changes in ESRD are structural rather than functional [20], that oxidative stress counteracts the effect of UF, or that the beneficial effect of acute volume reduction may be obscured by the activation of the rennin-angiotensin system [21, 22]. Other studies agreed that PWV remains unchanged as a more structural parameter [13, 14], while AI@75 decreases with the HD session (as a more functional one). This improvement has mainly been attributed to volume correction [12–15], although some authors have already questioned this association [16–18]. Given the lack of association with the UF, we analysed possible relationships with pre-HD brachial or aortic BP, pre-HD AI@75, BP decrease during HD or dialysis vintage, but we did not find correlation with any of these variables.

These discrepancies in the literature can be justified by two main reasons. On the one hand, the large number of variables that can influence during a HD session, not only immunoactivation and intravascular volume drop, but also BP changes, oxidative stress, activation of the rennin-angiotensin system, changes in calcium or magnesium levels during the session, or the differences between patients in intravascular refilling due to individual nutritional status, among others [21–26]. It is, therefore, very difficult to analyze each of them separately. On the other hand, there is a lack of uniformity in the way arterial stiffness is measured in the different studies.

We can conclude that HD procedure with the diacetate cellulose membrane induces a clear immunoactivation effect easily demonstrable by leukocyte cell count and C3a generation that is not detectable by PWA. The improvement of arterial stiffness observed after the HD session assessed by PWA was not related with ultrafiltration nor with changes in BP.

Further studies with control of the different variables should be carried out in order to determine how each of these factors affects arterial stiffness during the hemodialysis session.

## Figures and Tables

**Table 1:** Membrane bioincompatibility analysis (*n* = 11).

	Pre-HD	15 min	30 min	60 min	Post-HD	*P*
Immunoactivation						
Leucocytes	6801 ± 1186	4412 ± 1333	6062 ± 1528	6951 ± 1589	6648 ± 1238	<0.001
C3a (ng/mL)	427 ± 269	3501 ± 1638	2445 ± 1094	1915 ± 1009	953 ± 257	<0.000

Pulse wave analysis						
AI@75	26.1 ± 11.1	26.6 ± 12.4	23.5 ± 13.6	—	—	ns
ED	37.1 ± 5.9	35.8 ± 5.1	36.1 ± 4.4	—	—	ns
SEVR	129.1 ± 30.3	141.1 ± 29.5	138.5 ± 23.4	—	—	ns
Brachial SBP	135.1 ± 18.1	122.7 ± 27.4	125.8 ± 31.1	—	—	0.01
Brachial DBP	61.7 ± 7.5	60.6 ± 11.3	62.5 ± 11.1	—	—	ns
Central SBP	120.1 ± 17.7	110.4 ± 25.8	112.5 ± 30.9	—	—	0.009
Central DBP	63.1 ± 8.0	61.9 ± 10.9	63.4 ± 11.4	—	—	ns

The *P* value makes reference to the difference between pre-HD and 15-minute values.

AI@75: augmentation index corrected by heart rate; ED: ejection duration; SEVR: subendocardial viability ratio; SBP: systolic blood pressure; DBP: diastolic blood pressure.

**Table 2:** Ultrafiltration analysis (*n* = 19).

	Pre-HD	Post-HD	*P*
AI@75	29.9 ± 10.1	18.6 ± 15.0	<0.00
ED	37.6 ± 3.6	32.8 ± 4.6	<0.00
SEVR	124.6 ± 19.9	171.7 ± 37.1	<0.00
Brachial SBP	153.7 ± 26.7	136.0 ± 31.9	<0.00
Brachial DBP	74.7 ± 13.3	73.6 ± 17.6	ns
Central SBP	139.8 ± 25.5	119.4 ± 28.5	<0.00
Central DBP	75.8 ± 13.9	75.1 ± 17.9	ns

AI@75: augmentation index corrected by heart rate; ED: ejection duration; SEVR: subendocardial viability ratio; SBP: systolic blood pressure; DBP: diastolic blood pressure.
